# Restoration of Strength in Polyamide Woven Glass Fiber Organosheets by Hot Pressing: Case Study of Impact and Compression after Impact

**DOI:** 10.3390/polym16152223

**Published:** 2024-08-05

**Authors:** Mohammad Nazmus Saquib, Edwing Chaparro-Chavez, Christopher Morris, Kuthan Çelebi, Diego Pedrazzoli, Mingfu Zhang, Sergii G. Kravchenko, Oleksandr G. Kravchenko

**Affiliations:** 1 Department of Mechanical and Aerospace Engineering, Old Dominion University, Norfolk, VA 23529, USA; cmorr023@odu.edu; 2 Johns-Manville Corp., Denver, CO 80202, USA; edwing.chaparro-chavez@jm.com (E.C.-C.); diego.pedrazzoli@jm.com (D.P.); mingfu.zhang@jm.com (M.Z.); 3 Department of Materials Engineering, The University of British Columbia, Vancouver, BC V6T 1Z4, Canada; kuthan.celebi@ubc.ca (K.Ç.); sergey.kravchenko@ubc.ca (S.G.K.)

**Keywords:** low-velocity impact, high-velocity impact, 2/2 twill weave, thermoplastic (nylon) composite, damage tolerance, compression after impact (CAI), fusion bonding, crack healing, digital image correlation (DIC), micro-CT scan

## Abstract

Thermoplastic composite organosheets (OSs) are increasingly recognized as a viable solution for automotive and aerospace structures, offering a range of benefits including cost-effectiveness through high-rate production, lightweight design, impact resistance, formability, and recyclability. This study examines the impact response, post-impact strength evaluation, and hot-pressing repair effectiveness of woven glass fiber nylon composite OSs across varying impact energy levels. Experimental investigations involved subjecting composite specimens to impact at varying energy levels using a drop-tower test rig, followed by compression-after-impact (CAI) tests. The results underscore the exceptional damage tolerance and improved residual compressive strength of the OSs compared to traditional thermoset composites. This enhancement was primarily attributed to the matrix’s ductility, which mitigated transverse crack propagation and significantly increased the amount of absorbed energy. To mitigate impact-induced damage, a localized hot-pressing repair approach was developed. This allowed to restore the post-impact strength of the OSs to pristine levels for impact energies below 40 J and by 83.6% for higher impact energies, when OS perforation was observed. The measured levels of post-repair strength demonstrate a successful restoration of OS strength over a wide range of impact energies, and despite limitations in achieving complete strength recovery above 40 J, hot-pressing repair emerges as a promising strategy for ensuring the longevity of thermoplastic composites through repairability.

## 1. Introduction

Thermoplastic composites, reinforced with woven fabrics, have emerged [1,2] as highly sought-after materials in key industries such as automotive, aerospace, and energy owing to their efficient manufacturing process [3,4] and superior recyclability and repairability [5,6,7] compared to traditional thermosetting composites [8,9,10,11]. These composites offer numerous advantages, including unlimited shelf life, shorter processing times, and recyclability [12,13]. Unlike thermosets, thermoplastic composites can be processed at different heating and cooling rates without undergoing an exothermic reaction, while exhibiting higher toughness and lower shear and compression strengths [14,15]. Furthermore, composite structures are susceptible to impact damage, which can significantly affect their mechanical properties [8,9,16,17,18,19,20,21,22]. To address this challenge, repair processes are favored over replacement to restore strength, extend service life, and reduce maintenance costs [16,23,24,25,26,27]. Therefore, understanding impact-induced damage, evaluating residual properties, and developing effective repair strategies are crucial for structural composite materials.

Low- and high-velocity impacts on composites result in various damage modes such as matrix cracking, delamination, debonding, and fiber breakage [28,29,30]. Previous studies have highlighted matrix cracks and delamination as primary damage modes caused by low-velocity impacts [31,32,33,34]. Woven composites demonstrate reduced internal damage compared to unidirectional tapes due to the structural integrity of the weave, alongside offering high fracture toughness that mitigates matrix cracking [35,36]. Understanding post-impact properties is essential for designing new composite systems, as residual compressive strength and stiffness of composite structures diminish significantly compared to residual tensile properties due to impact-induced damage [37]. Various studies have explored the compression-after-impact (CAI) behavior of composites, emphasizing the significant influence of impact damage on compressive strength and behavior [38,39,40,41]. Effective repair methods are crucial for the widespread adoption and utilization of thermoplastic composites [20,24,42]. Repair methods for low-velocity impact damage in thermoplastic composites include patch repair, filler or plotting repairs, and fusion repair [43]. Fusion repair utilizes the fusibility of thermoplastics to restore strength by applying heat and pressure to the damaged region. While several studies have explored the impact behavior of woven composites and the repair of thermoplastic composites, further research is needed to fully understand and optimize their effective use in structural applications.

Research on polymer composites has predominantly focused on glass or carbon fiber-reinforced plastics (GFRPs or CFRPs), which are known for their excellent mechanical properties. However, these materials often use thermosetting polymer matrices, which present significant challenges in terms of cost and recyclability [44,45,46]. Recent advancements in engineering thermoplastic-based composites offer a promising alternative due to their lower cost and easier recyclability [45]. Studies have shown that these composites can absorb impact energy as well as, or even better than, CFRPs [46,47]. Additionally, they exhibit significantly higher impacts-to-failure in low-energy repeated impact tests, underscoring their potential for enhanced durability and cost-effectiveness. The development of advanced thermoplastic composites, which includes both high-performance and engineering thermoplastics, has gained attraction because of their advantageous properties. High-performance thermoplastics provide exceptional mechanical strength and impact resistance, while engineering thermoplastics offer a balanced combination of performance and cost-effectiveness [48]. Furthermore, thermoplastic composites can be fully restored after sustaining damage [23,24], unlike thermosetting composites which often require complete replacement. This reparability significantly enhances the appeal of thermoplastic composites for structural applications. 

Recent studies indicate a growing interest in thermoplastic composites reinforced with woven fabrics due to their superior impact resistance and damage tolerance [49,50,51]. Woven fabric reinforcement provides a balanced combination of strength, flexibility, and durability, making it suitable for a variety of structural applications. The interlaced structure of woven fabrics ensures uniform stress distribution and minimizes the risk of crack propagation and delamination [51]. Among various weaving architectures, twill weave is particularly notable for its advantages over plain weave, offering enhanced impact resistance and drapeability [52]. Interlacing fiber patterns, such as plain and twill weave architecture, facilitates better stress distribution and impact force dispersion, reducing localized damage and enhancing overall durability and perforation resistance [53,54]. These characteristics make woven fabric reinforcements highly advantageous for high-impact and durable applications in the automotive, aerospace, and defense industries. The combination of thermoplastic matrices with these advanced weaving architectures has the potential to offer promising solutions for next-generation, high-performance composite materials.

Despite significant advancements in understanding the impact behavior and repair mechanisms of thermoplastic composites, several gaps remain. Current research has predominantly focused on high-performance thermoplastics for the development of advanced materials as impact-resistant, durable structures [23,24,55]. However, this study aims to bridge this gap by investigating the impact and post-impact behavior of glass fiber woven fabric-reinforced polyamide, an engineering thermoplastic composite known as organosheet (OS) [56,57,58,59]. The OS, characterized by high ductility and the toughness of polyamide 6 (nylon 6) and the structural integrity of the twill woven fabric, exhibits exceptional impact resistance [16]. The research aims to assess its response to impact damage and propose a cost-effective localized hot-pressing repair approach to enhance durability. By evaluating the material’s behavior under impact and developing an effective repair strategy, this study aims to strengthen our understanding of the woven fabric-reinforced engineering thermoplastic composite response to impact events and provide valuable insights for future applications and repairs.

## 2. Materials and Methods

The impact and post-impact behavior of the woven OSs were comprehensively investigated through a series of tests. These included drop-weight impact tests, a subsequent hot-pressing repair procedure, flexural tests, and CAI tests. Impact-induced damage at various energy levels was assessed through the impact test, while the residual strength following impact was analyzed by the flexural and CAI tests. The compression and flexural tests conducted after repair provided valuable insights into the effectiveness of the localized hot-pressing technique in restoring strength.

### 2.1. Materials and Impact Test Specimens

**Figure 1:**
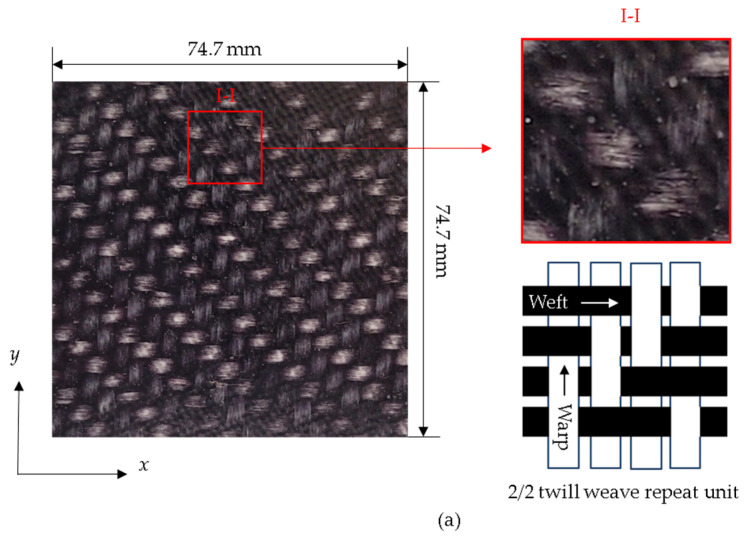

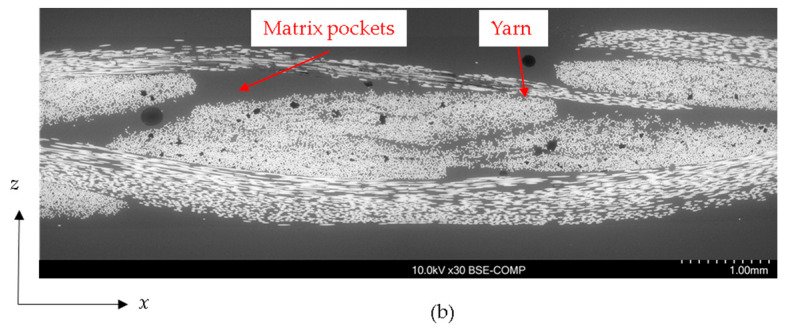
(**a**) The 2/2 twill woven OS test panel with illustration of local meso-structure. (**b**) Micrograph of OS cross-section.

The composite investigated in this study comprised a 2/2 twill woven glass fiber-reinforced polyamide 6 (PA6) matrix with a fiber content of approximately 44% by volume. These panels were sized at 74.7 × 74.7 × 1.8 mm using a waterjet and subsequently dried for 24 h in a Yamato DKN-400 convection oven at 85 °C. Post-drying, the specimens were conditioned for 48 h in a laboratory environment maintained at 23.3 °C and 50% relative humidity. The selected weave pattern, 2/2 twill, featured a repeated sequence of 2 horizontal yarns (weft) over and 2 yarns under (warp) [60]. Both warp and weft had an areal density of 2.25 yarns/cm, with a yarn density of 1200 g/m^2^, and the fibers within the yarns had a diameter of 16 µm. Figure 1 represents the investigated panel, showcasing the local meso-structure of the woven fabric and a micrograph of its cross-section.

### 2.2. Drop-Tower Impact Test

**Figure 2:**
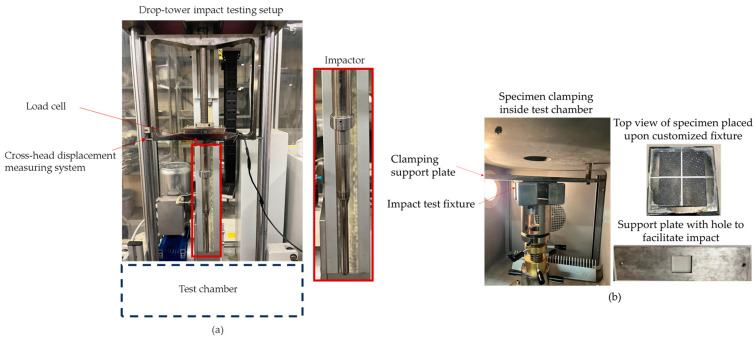

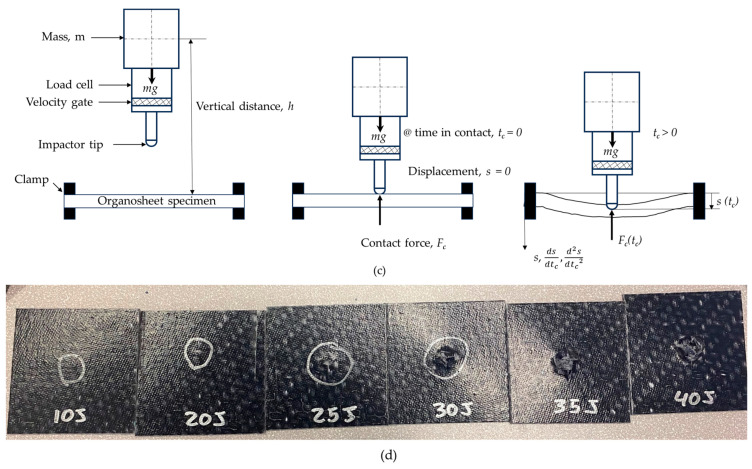
Drop-tower impact. (**a**) Test setup, and (**b**) specimen clamping system. (**c**) Schematic of three stages of impact test (modified from [62,63]). (**d**) Impact-induced damage at different impact energy levels.

Impact tests were performed using an Instron CEAST 9350 drop-weight testing system (Figure 2a), adhering to ISO 6603 standard testing protocols [61]. The setup involved out-of-plane impact loading on a flat woven composite plate. The load was applied by a free-falling impactor with a 12.8 mm diameter and 5.41 kg mass, released from a predetermined height, *h*. Before the test, the OS specimen was first aligned with the fixture by drawing straight lines down the middle of both the specimen and the fixture. Once aligned, the specimen was clamped inside the test chamber using a customized metal fixture and a support clamping plate, both featuring a 66 × 66 mm square hole (Figure 2b). The specimen was clamped under approximately 0.4 MPa force using the Instron program to prevent in-plane movements. Throughout the test, various parameters such as impactor velocity *v*(*t*), contact force *F_c_*(*t*), specimen displacement *s*(*t*), and energy applied to the specimen *E_app_*(*t*) were continuously recorded at a frequency of 2000 kHz. A pneumatic sensor in the test system helped to arrest rebound after impact. Figure 2c illustrates different stages of an impact event, and Figure 2d shows the OS specimens after impact.

### 2.3. Post Impact Hot-Pressing Repair

**Figure 3:**
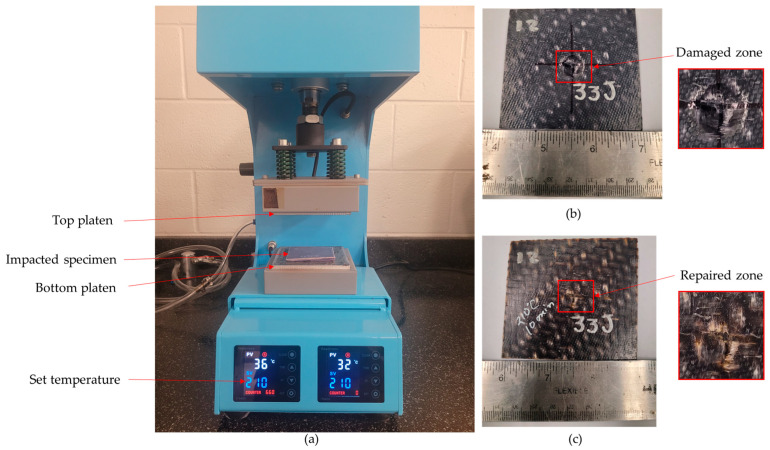
(**a**) Tabletop hot-pressing repair setup. (**b**) OS panel after impact damage. (**c**) OS after repair.

A localized hot-pressing technique using a tabletop hot press was developed to restore the strength of the OS following the impact damage in the woven PA6 OSs (Figure 3a). To protect the top surface of the OS samples during high-temperature healing treatment, Kapton tape was applied as a protective film before placing impacted OS samples into the hot press. The platens were set to a temperature of 210 °C, and the samples were preheated for 10 min. Subsequently, a pressure of 1.15 MPa was applied to the specimens for 30 min to facilitate the healing of the impact-induced damage. After the hot-pressing procedure, the samples were allowed to cool down before being removed from the press. Figure 3 illustrates an example of an impacted specimen before (Figure 3b) and after repair (Figure 3c). The zoomed-in views depict the damaged zone after impact damage and after hot-pressing repair.

**Figure 4:**
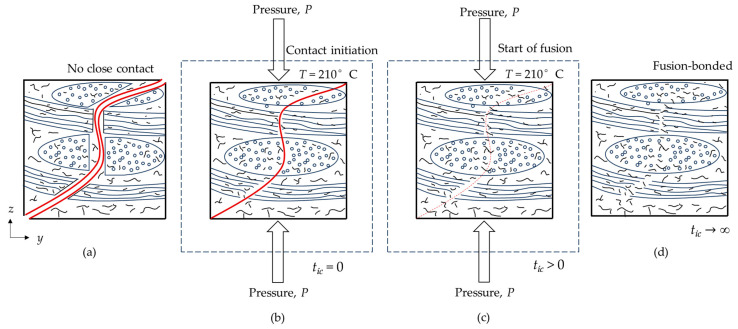
Illustration of stages during fusion bonding: (**a**) damaged surfaces are not in contact; (**b**) initial contact formation; (**c**) fusion initiation; and (**d**) fusion bonding of PA6.

The repair or bonding of two thermoplastic interfaces occurs when two surfaces in contact coalesce to form a single surface [64,65]. The resulting strength of the composite is a function of temperature, pressure, and time [66]. Two major factors affect the repair of a damaged thermoplastic composite by healing: close contact formation between the surfaces to be healed and macromolecule fusion across the surface in contact. Thermoplastic repair is governed by the formation of bonds between the cracked surfaces through the application of heat and the creation of close contact through pressure, a process known as fusion bonding or self-diffusion [67]. Fusion of interfaces is conditional, since healing requires surfaces to be in intimate contact, as molecules cannot travel in an open space [68]. The repair procedure for the woven OS started by heating the sample above its glass transition temperature (*T_g_*) [69] and applying adequate pressure (*P*) to ensure intimate contact (Figure 4b). For this study, we considered 1–2 MPa pressure, which was found to be sufficient, at these temperatures, to form close contact between the cracked interfaces to be healed [70]. As the temperature reaches above *T_g_*, the time for initial contact can be established (*t_ic_*) and the surfaces begin deforming viscoelastically. As a result, polymer chains start to diffuse across the surface boundaries [71] due to thermal motion (Figure 4c). At this stage, polymer chains entangle [72,73], crossing the interfaces and strengthening the structure (Figure 4d) and, thus, achieving the complete fusion bonding at $t_{ic}\to \infty$. Once this period is reached, the damage is considered to be healed [44,45,46,74,75], although damage to the fibers is irreversible.

### 2.4. Flexural Tests to Assess Fusion Bonding Repair Effectiveness

**Figure 5:**
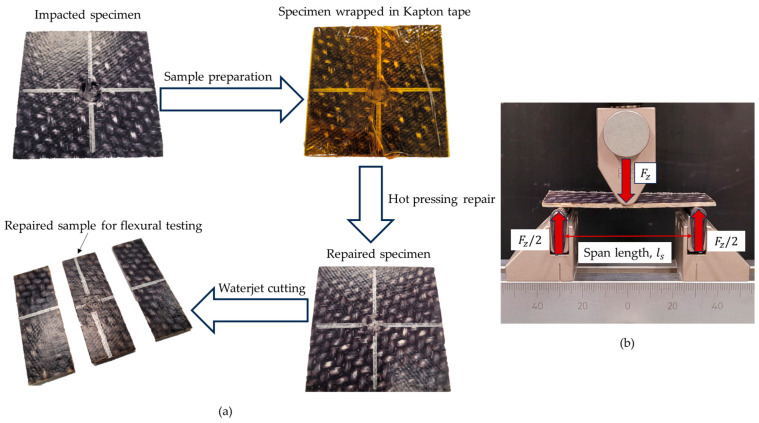
(**a**) Illustration of flexural test specimen preparation after repair. (**b**) Bending test setup used.

Flexural testing provided insight to assess the mechanical properties of pristine, impacted, and repaired OS samples. To prepare the flexural test specimens, the square samples (74.7 mm × 74.7 mm) post-impact were sectioned into three equal parts using waterjet cutting, as depicted in Figure 5a. The behavior of these specimens under bending loads was evaluated through flexural tests conducted in accordance with ASTM D7264 standards [76]. A standard three-point flexural test setup is illustrated in Figure 5b.

Flexural tests were conducted on both pristine and impacted specimens, as well as on repaired specimens subjected to impact damage at various energy levels and contact times, with temperature and pressure maintained constant to assess repair effectiveness and identify optimal repair procedures. During the flexural tests, data on flexural load (*F_z_*) and deflection (*δ_z_*) were recorded using an MTS machine, applying load at a rate of 2 mm/min. These *F_z_*-*δ_z_* data were then utilized to calculate the flexural stress (*σ_Flex_*), flexural strain (*ϵ_Flex_*), and flexural modulus (*E_Flex_*) using Equations (1)–(3) [77,78]:

(1) $$\sigma_{Flex}=\frac{3F_{z}l_{s}}{2wt^{2}}$$

(2) $$\epsilon_{Flex}=\frac{6\delta_{z}t}{{l_{s}}^{2}}$$

(3) $$E_{Flex}=\frac{{l_{s}}^{3}F_{z}}{{4w}_{s}\delta_{z}}$$

Here, *l_S_* represents the span length of the setup, set at 60 mm, while *w* and *t* denote the width and thickness of the sample, respectively. Flexural strength indicates the material’s ability to withstand bending stresses, whereas flexural modulus offers insights into its resistance to bending.

### 2.5. Compression-after-Impact Testing

**Figure 6:**
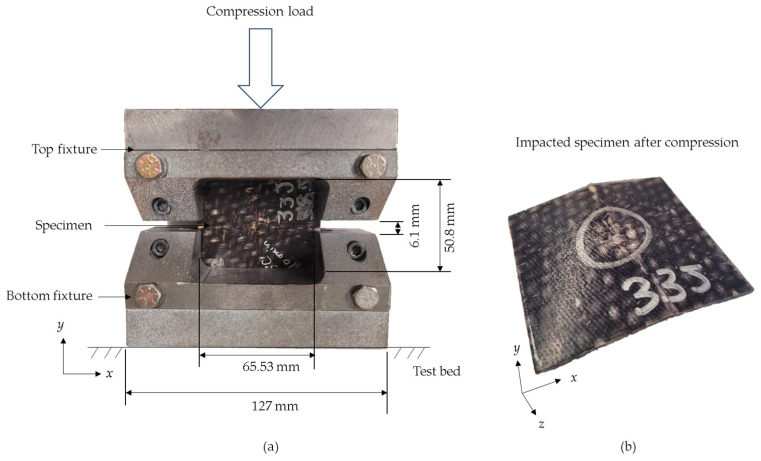
(**a**) CAI test setup. (**b**) Broken specimen after CAI test.

Compression testing (ASTM D7137) [79] was conducted on the pristine, impacted, and repaired specimens using an MTS test machine equipped with a 100 kN load cell (Figure 6). The tests were carried out under displacement control at a rate of 2 mm/min. The specimens were loaded in a CAI experimental setup, as illustrated in Figure 6a. Throughout the CAI testing, the reaction force (*F*) and crosshead displacement (δ*_z_*) were continuously monitored at a frequency of 5 Hz. The *F*-δ*_z_* curve obtained from these measurements enabled the determination of the failure force (*Fmax*), corresponding to the maximum force prior to failure. Subsequently, this value was used to calculate the compression residual strength (*σ_r_*) of the specimen, as per Equation (4) [63]:

(4) $$\sigma_{r}=\frac{F_{max}}{A}\;A=wt$$

where *A* represents the cross-sectional area of the specimen. Additionally, timed images were captured during the CAI testing at a frequency of 5 Hz using a digital image correlation (DIC) setup. These images were utilized for conducting failure analysis.

## 3. Results

### 3.1. Impact Response of the Woven OS Panel

**Figure 7:**
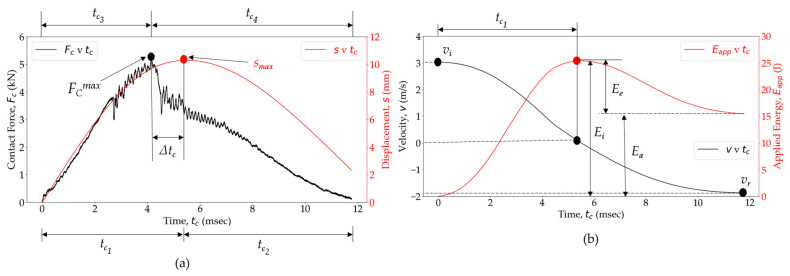
Impact behavior of woven OS at 25 J impact. (**a**) Contact force and displacement as a function of contact time. (**b**) Velocity and energy applied as a function of contact time.

During the impact event, deformation occurred in the local contact zone when the composite specimen and the impactor come into contact, resulting in an *F_C_* exerted by the specimen on the impactor. The effects of an impact event on woven glass fiber PA6 composites were investigated by analyzing variations in impact parameters such as *F_C_*, *t_c_*, s, *v*, and absorbed energy (*E_a_*). Figure 7 provides an overview of the impact responses of the studied woven composite panels, when subjected to a 25 J impact. As the impactor’s movement is opposed by the deforming composite specimen, its *v_i_* gradually decreases, accompanied by an increasing *F_C_*. The force–time (*F_C_*(*t_c_*)) curve obtained from the experimental measurements shows a stage of steady force increase over time until reaching the peak load (*F_C_^max^*). Smooth or low-amplitude fluctuations in the *F_C_*(*t_c_*) align with previous observations in various materials [62]. Temporary drops in the *F_C_*(*t_c_*) indicate the initiation and growth of local damage through meso-scale mechanisms, with the creation of new surfaces due to failure serving as an energy absorption mechanism during the impact event [80,81]. For non-perforated specimens, the *v* decreases over time and reaches zero when the *s* reaches its maximum value for the given impact energy. The impactor then starts its return movement, accelerating away from the specimen. Comparing the *F_C_*(*t_c_*) and *s*(*t_c_*) curves, it is observed that it takes more time to reach maximum displacement (*s_msn_*) compared to *F_C_^max^*. The impactor continues to move downward to some extent after reaching the *F_C_^max^*, causing a time delay Δ*t_c_* between *F_C_^max^* and *s_max_* due to the development of damage mechanisms in the material. These mechanisms lead to different downward travel (*t_c_*_1_) and rebound times (*t_c_*_2_) for the impactor, as well as loading (*t_c_*_3_) and unloading times (*t_c_*_4_). Pure linear elastic contact results in *t_c_*_1_ = *t_c_*_2_ = *t_c_*_3_ = *t_c_*_4_ and Δ*t_c_* = 0 (Hertzian solution) [63]. Inelasticity in an impact event results in variations in these times.

As the impactor’s kinetic energy is transferred to the composite specimen during its downward movement, elastic strain energy is stored, leading to an increasing *E_app_*(*t_c_*) curve. Once the local material strength is reached, a portion of this stored energy dissipates through irreversible damage. When all the incident kinetic energy (*E_i_*) of the impactor is transferred to the composite specimen, the *E_app_*(*t_c_*) curve peaks at the same time when the impactor’s velocity approaches zero at *s_max_* = 0. Subsequently, the *E_app_*(*t_c_*) curve gradually decreases as stored energy, accumulated in the composite specimen, is transferred back into the impactor, causing it to rebound. Finally, the *E_app_*(*t_c_*) curve reaches a constant value, representing the total energy permanently absorbed by the composite specimen at the end of the impact event, which is not restituted to the rebounding impactor. The part of the *E_i_* responsible for the impactor to bounce back is known as elastic strain energy, *E_e_*. More specifically, *E_a_* results from damage mechanisms and includes contributions from other non-conservative phenomena such as vibration, damping, friction, and specimen/fixture slipping [63].

**Figure 8:**
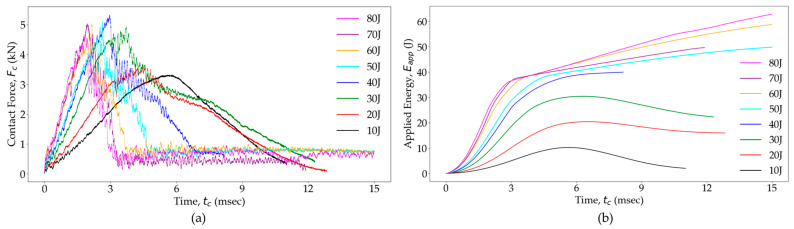
(**a**) Transient load and (**b**) energy response for the OS at different impact energy levels.

**Figure 9:**
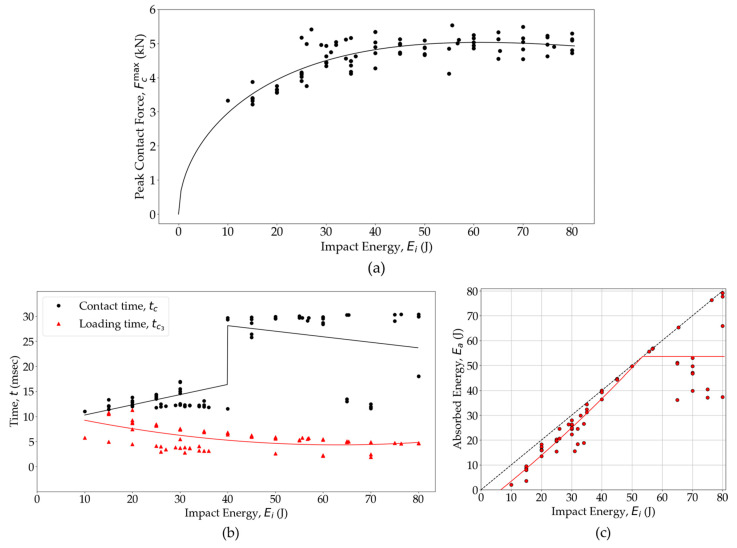
Comparison of (**a**) peak force, (**b**) contact time and time to reach peak load, and (**c**) absorbed energy against impact energy for the OS panels.

Figure 8 presents the *F_C_*(*t_c_*) and *E_app_*(*t_c_*) curves for the glass fiber/PA6 woven OS at various energy levels. The *F_C_*(*t_c_*) curve (Figure 8a) shows how the OS samples responded structurally to impacts at different energy levels. As the impact energy increased, *F_C_^max^* also increased, and the time to reach *t_c_*_3_ decreased. Similarly, higher energy impacts resulted in longer rebound times, *t_c_*_4_. This increased *t_c_*_4_ was attributed to faster damage initiation and prolonged damage growth at higher energy levels. However, impacts exceeding 40 J displayed reduced *F_C_^max^* (Figure 9a), which stems from a decrease in the local flexural stiffness caused by increased damage [82], making the specimen more compliant [83]. Consequently, a reduced *F_C_^max^* and prolonged *t_c_* (Figure 9b) were observed when the effective structural stiffness of an impacted specimen was reduced due to greater damage accumulation.

The *E_app_*(*t_c_*) curves (Figure 8b) demonstrate how energy was transferred between the impactor and the composite specimen during the impact event. The maximum energy value in the curve represents *E_i_*, indicating the total energy introduced into the specimen. Following the contact and rebound phases, the energy value equates to *E_a_*. For impacts with energy levels below 20 J, *E_a_* is considerably smaller than *E_i_*, indicating minimal energy dissipation during the impact event. In such cases, the *E_e_* within the specimen causes the impactor to rebound with higher velocity (*v_r_*). However, as the impact energy exceeds 20 J, energy dissipation through damaged surfaces becomes more prominent, leading to a decrease in the *v_r_* of the impactor. Notably, impacts exceeding 40 J result in *E_a_* becoming equal to *E_i_* over time, indicating that all of the energy is absorbed by the composite specimen.

As observed in Figure 9a, the peak contact force does not increase monotonically as the *E_i_* increases, but instead reaches a plateau asymptotically, concurrent with the accumulation of damage. The data presented in Figure 9a affirm that the composite panels reach a dynamic peak force plateau for *E_i_* > 40 J, when perforation of the OS sample is observed (Figure 2d). When an *F_C_^max^* data scatter plateaus, it maintains a consistent mean value for *F_C_^max^* regardless of increasing *E_i_* [63]. The experimental data in Figure 9a were a fitted *F_C_^max^*(*E_i_*) regression curve utilizing a previously proposed spring–mass–dashpot model [83,84] outlined in Equation (5), wherein *a*, *b*, and *c* serve as the fit coefficients.

(5) $${F_{c}}^{max}\left(E_{i}\right)=-\frac{a}{2}{\left(\frac{2E_{i}}{1.73}\right)}^{\frac{b}{2}}+\sqrt{2cE_{i}+2^{b-2}\frac{a^{2}}{1.73^{b}}{E_{i}}^{b}}$$

The *F_C_^max^*(*E_i_*) plot indicates that the majority of impact tests conducted on woven OS samples effectively reside in the region of dynamic plateau. Here, as the impact energy increased, the local damage size increased, yet the *F_C_^max^* remained constant, which is indicative of the impactor penetration, as reflected in the perforation of the sample.

Figure 9b illustrates the relationship among *t_c_*, *t_c_*_3_, and *E_i_*. The *t_c_* increases with *E_i_* up to 40 J. Beyond this threshold, while *t_c_* generally continues to increase with *E_i_*, there are instances where *t_c_* decreases. These decreases in *t_c_* with increasing *E_i_* are attributed to perforation events [85]. Additionally, the *t_c_*_3_ shows a decreasing trend with increasing *E_i_* until it reaches a plateau around the penetration energy threshold of 40 J. This trend is typically observed when *t_c_*_3_< *t_c_*_4_ [81,82,86].

The diagram illustrating the relationship between *E_i_* and *E_a_*, commonly referred to as an “energy profile [81,87]” or “energy absorption curve [88]”, serves as an important way for understanding the impact behavior of composite materials [86]. In Figure 9b, energy profiles for the tested woven composite specimens are presented, alongside a diagonal line representing equal energy between impact and absorption. When *E_a_* is minimal, it suggests that the damage inflicted on the specimen during impact is minor, with most of the energy stored elastically in the specimen, to be later converted back into kinetic energy during impactor rebound. Previous studies have noted a quadratic relationship [88,89] between *E_a_* and *E_i_*, indicating that as *E_i_* increases, *E_e_* used for impactor rebound decreases. Equality between *E_i_* and *E_a_* signifies complete absorption of impact energy by the specimen, defining the penetration threshold. As *E_i_* increases more, the penetration process may lead to specimen perforation, after which absorbed energy and peak contact force remain nearly constant despite further increases in impact energy. This indicates that once perforation occurs, the impactor does not cause additional damage to the specimen even with increased impact energy. Figure 9b shows, below a 20 J impact, that *E_a_* is consistently less than *E_i_*, which suggests minor damage accumulation. Beyond a 25 J impact, the OS exhibits an increase in internal damage in some test panels, absorbing approximately 90% of incident energy. Above a 35 J impact, some data points start to align with the diagonal line, indicating full absorption of *E_i_* by the OS, which is a sign of penetration. Beyond 50 J, the fitted line accounts for perforation as some data showed no increases in *E_a_* with increasing *E_i_*. However, it is important to note that data scatter increases at higher energy levels, indicating variability in the response of the OS to impacts of greater magnitude. As the impact energy surpasses 50 J, some samples show instances of perforation with limited energy absorption, which is a common behavior for composites [50]. However, some OS samples also exhibit complete absorption of incident energy even at an 80 J impact, which can be attributed to the significant fracture toughness and ductility of PA6-based woven glass fiber OS. Furthermore, variations in the local meso-structure of the OS at the impact point are expected to contribute to data scatter. This suggests the potential for further study on the relationship between the local meso-structure and impact energy absorption, as the local meso-structure has been found to impact the tensile and open-hole tensile strength of woven OS [90].

**Figure 10:**
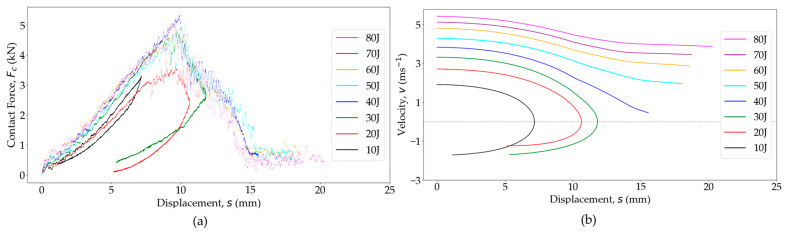
(**a**) Contact force–deflection and (**b**) impactor velocity–deflection curves for the woven OS test panels at various impact energy levels.

The force–displacement curves *F_c_*(*s*) serve as key indicators of how a composite material responds under impact loading [81], as shown in Figure 10a. It is important to note that the impactor displacement includes both the displacement of the specimen and the local indentation [80]. At an impact energy of 10 J, the *F_c_*(*s*) curve forms a closed loop, where the descending *F_c_* corresponds to decreasing *s*, suggesting the impactor is rebounding without external damage. In undamaged specimens, the hysteresis observed in the loading and unloading phases of an *F_c_*(*s*) curve signifies energy dissipation during the impact event. The area under the curve represents the deformation energy transferred from the impactor to the test panels and from the test panels to the rebounding impactor. The enclosed area within the loop represents absorbed energy, *E_a_*, during impact, with a larger area indicating greater *E_a_*. For impacts ≥ 20 J, the *F_c_*(*s*) curves no longer exhibit a closed loop due to energy dissipation through local damage and plasticity. At impacts ≥ 40 J, the *Fc*(*s*) curves are no longer closed, and displacements increase with decreasing loads, indicating penetration, resulting in reduced *E_e_*, which is transferred back to the impactor. Another noteworthy observation is that the slope of the *F_c_*(*s*) curve increases with rising impact energy due to the strain rate-dependent elastic behavior of PA6 [86]. Furthermore, the non-linear behavior of the *F_c_*(*s*) curves within a loading portion, along with significant deflection, which is several times the specimen’s thickness, suggests membrane stiffening and, as a result, the involvement of specimen membrane in-plane strength in the damage process development [80,91].

Figure 10b presents a comparison of the velocity variation with displacement, *v*(*s*), for the woven OS test panels subjected to various impact energies. For non-penetrated specimens (<40 J impact), the *v* initially peaked at the contact initiation, then gradually decreased in a parabolic manner to zero at *s_max_*, indicating the impactor’s rebound. Subsequently, the velocity vector reversed direction, with its magnitude increased as *s* decreased, reaching a near-constant value, signifying the impactor’s rebound. The rebound or exit velocity, *v_r_* at the end of the contact, is contingent upon *E_a_* during the impact event. At a 40 J impact, penetration indications emerged as *v* approached zero at *s_max_*, suggesting complete energy absorption through damage. Post-penetration (>40 J), the *v*(*s*) curve became non-parabolic, plateauing at *s_max_*, and *v_r_* became non-negative, indicating exit with reduced *v* after perforation.

**Figure 11:**
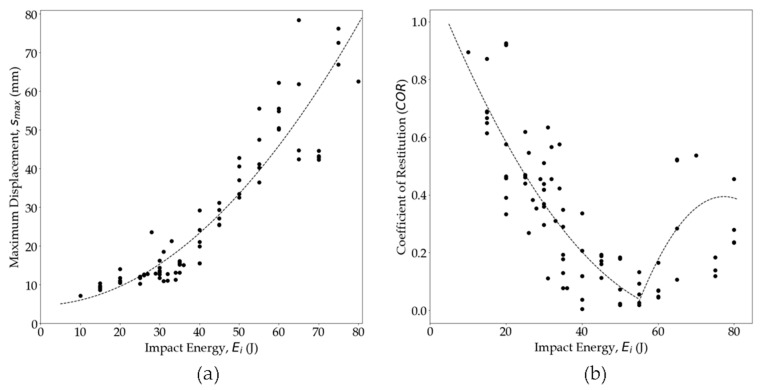
(**a**) Maximum displacement and (**b**) coefficient of restitution comparison at different impact energies [The dashed line represents curve fitting for the experimental dataset].

The analysis of *F_C_*(*s*) for increasing *E_i_* (Figure 10a) revealed a significant increase in *s_max_* until the penetration point. This illustration shows that *s_max_* increased with *Ei* until penetration and experienced a sudden jump around the penetration point. After perforation, *s_max_* progression stabilized again, with no expected sudden jumps [85]. Figure 11a illustrates that the *s_max_* of the impacted surface increased with the increase in *E_i_* up to the perforation point (<40 J). However, above a 45 J impact, the data exhibited scattered *s_max_* values for a given *E_i_*. A quadratic curve-fitting line is included to fit the data. Data points well below the line represent the perforated panels where *s_max_* did not increase as much with *E_i_*. Some instances showed much higher *s_max_* with increasing *E_i_*, indicating high energy absorption due to PA6 ductility.

The ratio of the *v_r_* to *v_i_*, known as the coefficient of restitution (*COR*) (Equation (6)), serves as a critical parameter in evaluating the impact behavior of materials [81,89].

(6) $$COR=\left|\frac{v_{r}}{v_{i}}\right|=\sqrt{\frac{\left(E_{i}-E_{a}\right)}{E_{i}}};0\;\leq COR\;\leq 1$$

A *COR* of 1 indicates a perfectly elastic impact event, while a *COR* of 0 suggests complete energy loss of the impactor. As the *E_i_* increases, the *COR* decreases due to greater energy dissipation resulting from increased damage to the specimen. At penetration, the *COR* reaches 0. However, after perforation, the impact energy exceeds the specimen’s capacity to absorb or dissipate, leading to an increase in *COR* with higher impact energies [52]. Figure 11b illustrates the relationship between *COR* and *E_i_*. As the *E_i_* increases, the *COR* decreases until perforation. At a 40 J impact, the *COR* drops down close to 0, suggesting significant damage and penetration. Some test panels above a 50 J impact had a *COR* starting to increase again, with *E_i_* indicating perforation.

### 3.2. Post-Impact Strength Evaluation

**Figure 12:**
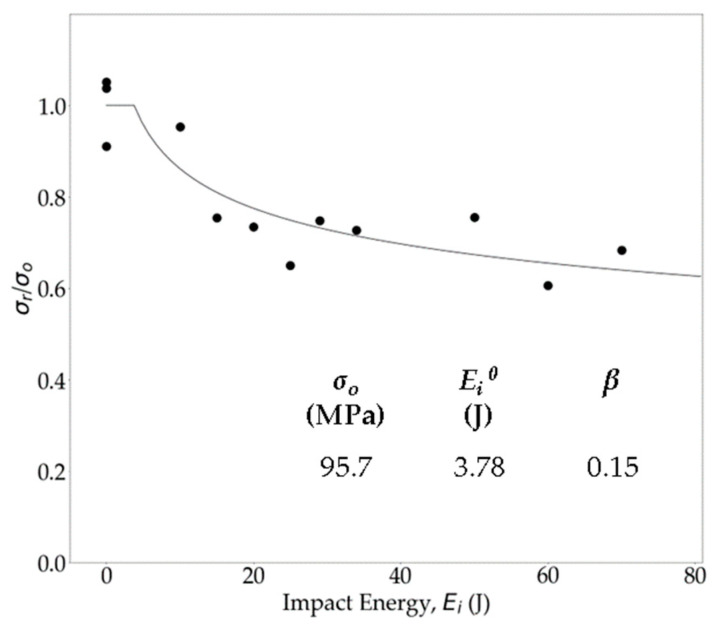
Experimental scatters and curve fitting of normalized CAI strength at different impact energy levels.

CAI tests are standard for evaluating the residual strengths (*σ_r_*) of impacted OS samples. These results are analyzed by plotting the normalized residual strength ($\frac{\sigma_{r}}{\sigma_{0}}$) against *E_i_*, where *σ*_0_ is the unimpacted strength of the panel. To establish reference values and quantify the strength drop after impact, *σ*_0_ (95.7 MPa) was measured using compression testing of three pristine OSs. Figure 12 presents a comparison of the normalized CAI residual strengths at different *E_i_*. While the CAI test results display some experimental scatter, they were fitted using a curve-fitting equation (Equation (7)), commonly employed for CAI data analysis [92].

(7) $$\frac{\sigma_{r}}{\sigma_{0}}={\left(\frac{{E_{i}}^{0}}{E_{i}}\right)}^{\beta }$$

Here, *E_i_*^0^ and *β* are the fit parameters, with *E_i_*^0^ representing the impact energy above which the compressive strength reduction begins for impacted specimens. The values of these fit parameters are reported in Figure 12. The results indicate that the OS demonstrated high damage tolerance, with no strength reduction observed until the 3.78 J (*E_i_*^0^) impact. However, as *E_i_* increased, a downward trend in *σ_r_* was observed, indicating an increase in damage propagation. This heightened damage resulted in a decrease in *σ_r_* of the specimens. Significantly, a substantial drop in strength (30–37%) was observed above a 40 J impact, highlighting the impact-induced damage in the specimen. Despite this notable reduction in *σ_r_*, the OS exhibited higher resistance to impact compared to thermoset polymer matrix composites [41,93].

**Figure 13:**
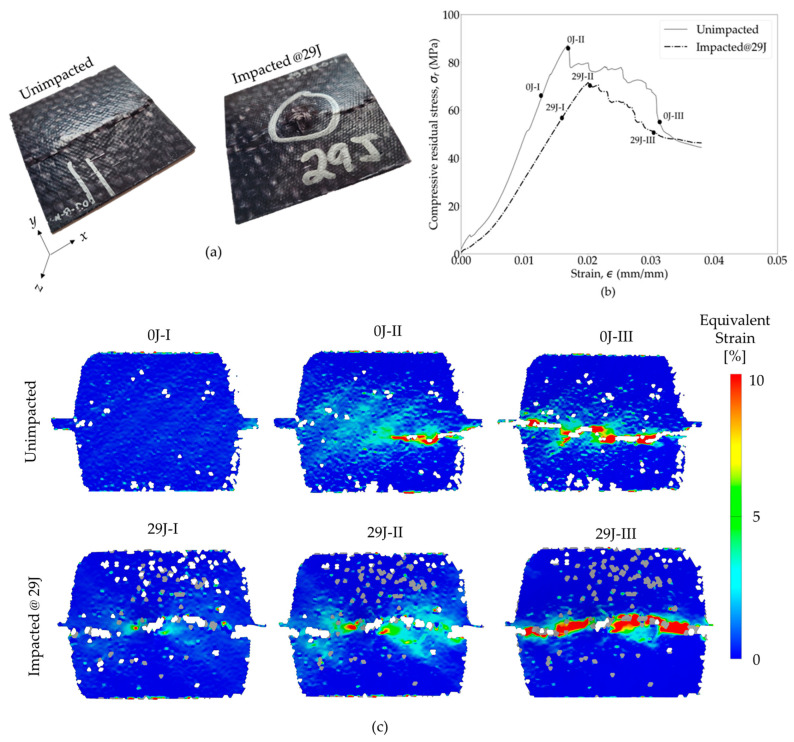
(**a**) Rear view of the pristine and impacted (29 J) specimens after undergoing compressive loading, (**b**) compressive stress–strain curves, and (**c**) progression of DIC strain field for unimpacted and impacted OSs during CAI tests [I, II and III represent stages before ultimate strength, at ultimate strength, and after failure, respectively].

During the compressive loading of both impacted and pristine specimens, DIC images were captured to facilitate a post-mortem analysis (Figure 13a). The comparison of the DIC strain field progression on the surfaces of specific unimpacted and impacted (29 J) specimens is presented in Figure 13c, while the corresponding compressive stress–strain curves are highlighted in Figure 13b. The *σ_r_*(ϵ) curve shows a reduction in strength and stiffness in the impacted specimen. However, the failure strain observed was in the similar range, indicating no loss of ductility due to impact. For the DIC analysis, in the case of the pristine sample, buckling occurred during failure, and there was no discernible crack propagation visible on the surface. The DIC strain field revealed an evolution from one side of the specimen, progressing through the middle to the other side. In contrast, for the impacted specimen, damage development during compressive loading initiated and propagated from the initial impact damage site. However, the impacted specimen also failed due to buckling, which is common for a thermoplastic resin woven composite [41]. The enhanced damage tolerance and increased compressive *σ_r_* of the OS was attributed to the ductility of the PA6 and the twill weave geometry of the yarn. PA6’s ductility played a pivotal role in reducing transverse crack propagation within the specimen, thus strengthening its damage tolerance capacity and maintaining flexural stiffness. Moreover, the woven fabric structure effectively countered through-thickness damage growth, thereby further enhancing the OS’s resistance to impact with a high strength level.

### 3.3. Repair Effectiveness Assessment

Internal damage resulting from impact events exceeding 20 J induced a notable reduction in the compressive strength of the woven OS meso-structure. To address this issue, a localized heating technique was employed as a method of hot-pressing repair. Different contact times were utilized during the repair process to determine an optimal repair procedure. Subsequently, the effectiveness of this repair technique was assessed through both flexural and CAI tests.

#### 3.3.1. Effect of Hot-Pressing Time on Impact Repair

The localized hot-pressing repair of the impacted specimens, all impacted at the same *E_i_*, was conducted at three different *t_ic_*: 10, 30, and 60 min. Throughout the repair process, temperature (210 °C) and pressure (1.15 MPa) were maintained constant to assess the influence of *t_c_* on the fusion bonding of PA6 OSs. The duration of the repair process holds significance as a longer *t_c_* may facilitate enhanced interdiffusion of the polymer chains at the interfaces, potentially resulting in stronger bonding [94]. Conversely, excessively prolonged *t_c_* durations could induce material degradation due to prolonged heat exposure, compromising the bond strength of repair [95,96].

**Figure 14:**
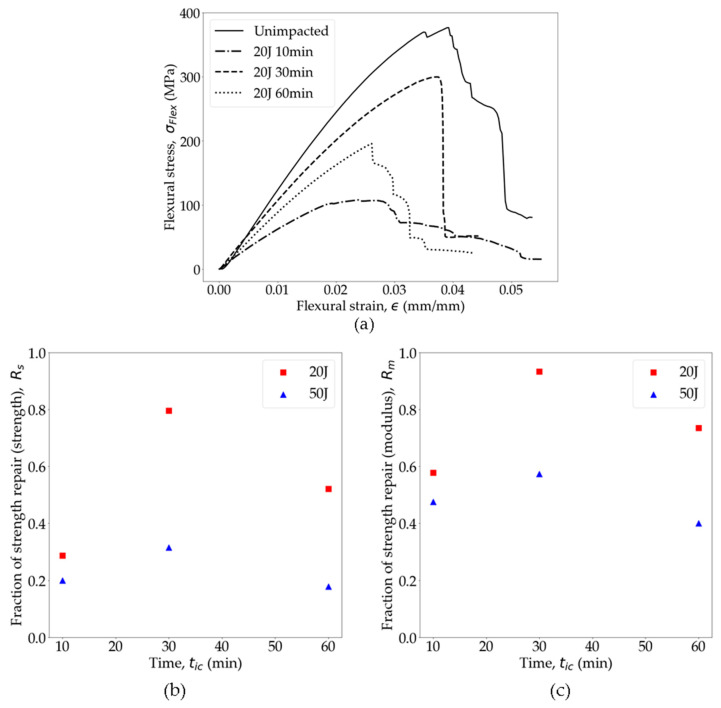
(**a**) Comparison of repaired OSs at different contact times. Repair fractions of (**b**) strength and (**c**) modulus at different times.

The effect of *t_ic_* on the repair of the OS was evaluated by flexural testing of the unimpacted, impacted (20 J and 50 J), and repaired specimens. In Figure 14a, the flexural stress–strain curves of the repaired specimens at different *t_ic_* are compared with the unimpacted specimens. Notably, the specimen hot-pressed for 30 min exhibited the most favorable results in terms of flexural strength and modulus, with a recovery of almost 80% of the pristine strength and more than 95% of the pristine modulus. Essentially, the fusion bonding of PA6 during hot pressing contributed to the recovery of both modulus and strength. However, it is important to note that the damaged fibers could not be restored, thus limiting complete recovery. Evidence suggests that specimens hot-pressed for 10 min did not achieve sufficient fusion bonding strength, resulting in significantly lower strength and modulus compared to pristine OSs. Allowing polymer chains to diffuse between cracked surfaces for a longer duration improved bonding efficacy. However, hot pressing the specimens for 60 min at 210 °C, significantly exceeding its *T_g_* and nearing its *T_m_*, exhibited signs of material degradation [96,97] and loss of fiber alignment resulting in both the strength and modulus being reduced compared to the 30 min duration. Furthermore, the PA6 crystallinity is likely increased during 60 min of pressing, leading to a loss of ductility and a reduction in fracture strain [70,98,99].

The mechanical properties of the repaired specimens were further assessed to determine the degree to which the specific samples were repaired by finding fraction of strength repair with respect to flexural strength and modulus, *R_s_* and *R_m_* (Equations (8) and (9)), following Wool’s crack healing theory of thermoplastics [75].

(8) $$R_{s}=\frac{\sigma_{r}(t_{ic})}{\sigma_{0}}$$

(9) $$R_{m}=\frac{E_{r}(t_{ic})}{E_{0}}$$

Here, $E_{0}$ and $E_{r}$ are the unimpacted and repaired modulus of the specimens, respectively. Figure 14b shows the trends of *R_s_* and *R_m_* as a function of *t_ic_*. For both the 20 J and 50 J impact-damaged samples post-repair, similar trends of *R_s_* and *R_m_* were observed. Following these trends, it can be inferred that the optimal *t_ic_* for repairing PA6 OSs should fall within the range of 30–40 min of hot pressing for the specified temperature and pressure. Consequently, the repaired test specimens utilized in this study underwent a 30 min repair process. Additionally, it was observed that the *R_s_* and *R_m_* for the 50 J impact-damaged specimens were significantly lower compared to the 20 J specimens. For the 20 J impacts, minimal fiber damage is anticipated as the impact energy is below the perforation threshold. Consequently, the damage is predominantly in the form of PA6 cracks, which can be effectively repaired through fusion bonding. However, for impacts above the perforation threshold, the damage extends to both the fiber and matrix. Fiber damage, in particular, is more detrimental to the overall structural integrity. As a result, hot pressing at this level of damage is expected to be less effective in restoring the original strength and modulus compared to the 20 J impacts.

#### 3.3.2. Micro-Computed Tomography (µ-CT) Scan of the Repaired Specimens

The nature and extent of the impact-induced damage, as well as the effectiveness of the repair, were examined using micro-computed tomography (µ-CT) scanning. OS samples subjected to 25 J and 33 J impacts and subsequent repair were selected for scanning with the SKYSCAN 2214 scanner. The scans were performed at a voltage of 50 kV and a current of 75 µA, achieving a resolution of 20–21 μm without any additional filter. The resulting raw images were analyzed for visualization using the VGSTUDIO MAX 2024.1 software.

**Figure 15:**
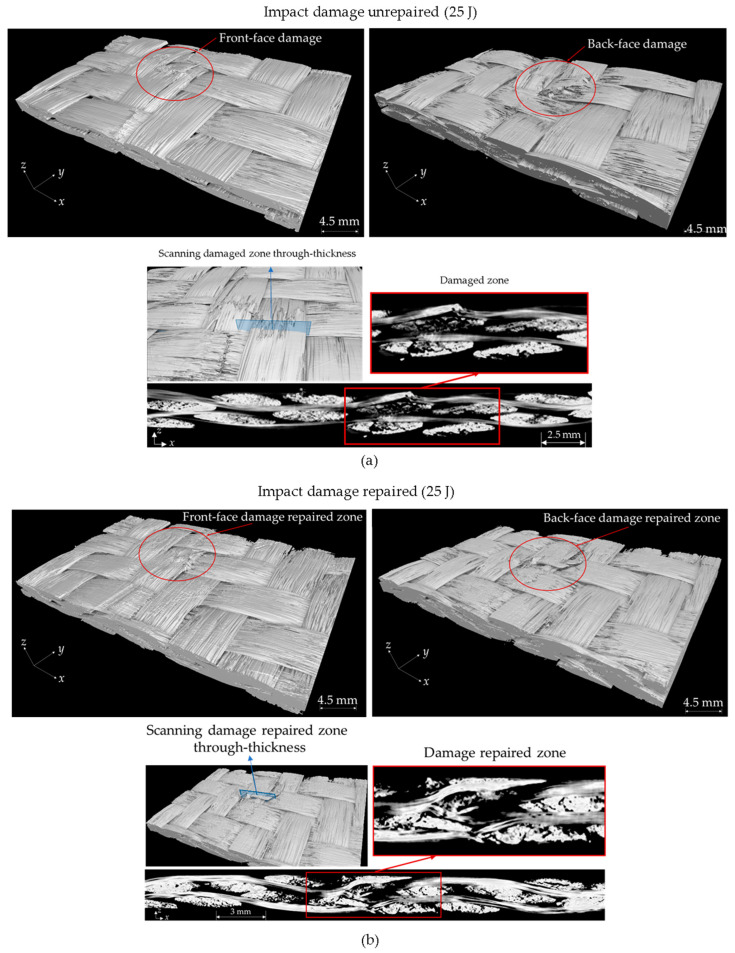

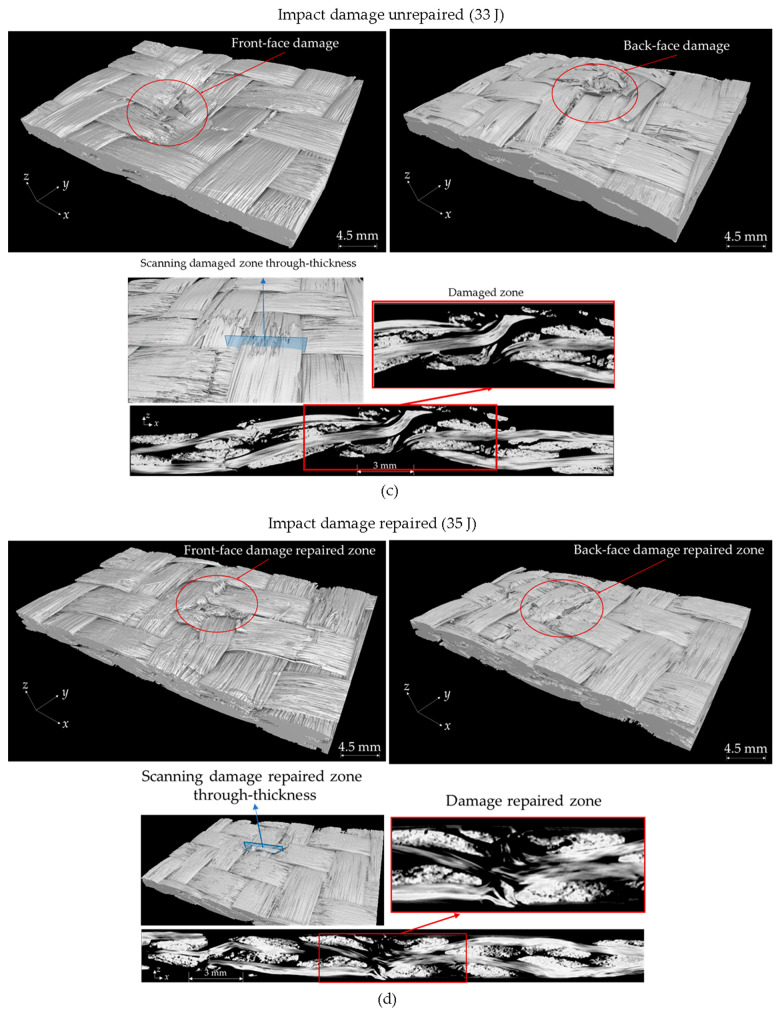
µ-CT cross-section of weave OS specimen after (**a**) 25 J, (**c**) 33 J impact and subsequent (**b**,**d**) repairs.

Figure 15 presents µ-CT images of the impacted and subsequently repaired samples, offering a detailed view of the damage morphology and the repair zone. The extent of the observed damage is both complex and three-dimensional. Figure 15a,c show the front-face, back-face, and cross-sectional views of the woven OS after impact. Various damage modes are evident, including through-thickness damage and fiber rupture. Following the impact, this damage creates significant structural weaknesses. However, after hot-pressing repair, as illustrated in Figure 15b,d, the frustum cone-shaped (Figure 15c) through-thickness damage caused by the impact, which is typical for continuous fiber composites [85,86,87], was recovered to some extent by realigning the broken fibers. The hot-pressing process facilitated the healing of matrix cracks through fusion bonding. The applied pressure and elevated temperatures allowed for the matrix to flow, while applying external pressure realigned the broken fibers, effectively restoring the material’s structural integrity.

#### 3.3.3. Post-Impact Strength Restoration

**Figure 16:**
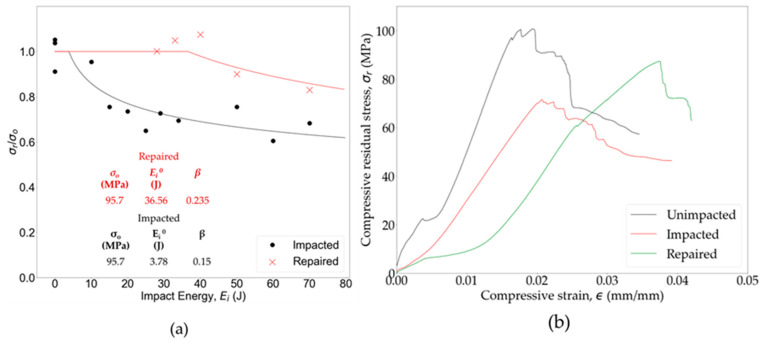
Evaluation of hot-pressing repair effectiveness: (**a**) CAI test results and (**b**) bending test results for an impacted and a repaired (*t_ic_* = 30 min) specimen at 29 J.

The impact-induced damage resulted in a 20–40% reduction in strength for impacts ranging from 20 J to 80 J. Figure 16a illustrates the comparison of compressive strength between the repaired samples (*t_ic_* = 30 min) and those with impact damage. The analysis reveals that the application of localized heating for hot pressing led to the restoration of the pristine compressive strength in the PA6 OS panels for impact levels below sample perforation. Remarkably, even in cases where the woven OS had undergone a 33 J impact, the compressive strength was successfully restored to its original level. Curve fitting of the CAI scattered data using Equation (7) indicates that complete strength recovery is possible up to a 36.56 J (*E_i_*^0^) impact. Figure 16b presents the assessment of repair effectiveness through a comparison of CAI test results among a pristine sample, a sample impacted at 29 J, and subsequently repaired sample. The findings reveal that the OS panel experienced a 28.93% reduction in flexural strength following a 29 J impact. However, employing localized hot-pressing repair to the similar energy-level-impacted sample enabled it to regain more than 86% of its original strength.

At impact levels above perforation, beyond a 40 J impact, complete compressive strength recovery was no longer achievable, which as shown previously is due to substantial fiber damage. Despite this limitation, hot-pressing repair was able to restore 83% of the pristine strength at an impact as high as 70 J. This restoration was achieved by utilizing the fusion of the damaged thermoplastic PA6 within the resin-rich pockets and inside of the glass fiber tows, which enabled effective healing at the selected high temperatures and pressures for the repair. Furthermore, recovery of planar orientation of the damaged fibers (Figure 15) contributed to the restoration of OS strength after impact. Figure 16b presents the assessment of the repair effectiveness through a comparison of the CAI test results among a pristine sample, a sample impacted at 29 J, and a subsequently repaired sample. The findings reveal that the OS panel experienced a 28.93% reduction in flexural strength following a 29 J impact. However, employing localized hot-pressing repair to the similar energy-level-impacted sample enabled it to regain more than 86% of its original strength.

## 4. Conclusions

In conclusion, this study delved into the impact response, post-impact strength evaluation, and repair effectiveness of woven glass fiber PA6 OSs subjected to varying impact energy levels. An analysis of the impact response unveiled intricate behaviors, including force–time and energy–time curves, elucidating the material’s exceptional energy absorption capability. Through CAI tests, a reduction in compressive strength was observed following impacts, notably for energy levels surpassing 10 J.

To mitigate these issues, a localized heating technique for hot-pressing repair was employed, assessing different contact times to determine optimal repair procedures. Notably, the investigation revealed that a 30 min repair process at 210 °C and 1.15 MPa yielded the most favorable outcomes, with strength and modulus recovery reaching 80% to 95% of pristine levels depending on the impact energy. The pristine compressive strength of the organosheet was 95.7 MPa. Curve fitting of the CAI scattered data showed no strength loss until a 3.78 J impact, and complete strength recovery is possible up to a 36.56 J impact by hot-pressing repair. At a 40 J impact, the OS panel experienced a 28.93% reduction in strength. However, employing localized hot-pressing repair to similarly impacted samples enabled them to regain more than 86% of their original strength.

Micro-CT scans provided valuable insights into the repair process, while post-repair strength evaluation confirmed successful restoration of compressive strength to pristine levels, particularly for impacts below perforation, which occurred at 40 J. Although complete strength recovery was challenging at higher impact energies, hot-pressing repair demonstrated the potential to substantially restore mechanical properties. These results underscore the promising avenues for enhancing structural integrity and durability of the woven thermoplastic OSs in practical applications, with further research aimed at refining repair techniques and exploring their applicability in real-world scenarios.

## Data Availability

The data presented in this study are available on request from the corresponding author. The data are not publicly available due to the proprietary model.
